# Authors’ Response to Peer Reviews of “Financial Feasibility of Developing Sustained-Release Incrementally Modified Drugs in Thailand’s Pharmaceutical Industry: Mixed Methods Study”

**DOI:** 10.2196/77623

**Published:** 2025-07-01

**Authors:** Manthana Laichapis, Rungpetch Sakulbumrungsil, Khunjira Udomaksorn, Nusaraporn Kessomboon, Osot Nerapusee, Charkkrit Hongthong, Sitanun Poonpolsub

**Affiliations:** 1 Department of Social and Administrative Pharmacy, Faculty of Pharmaceutical Sciences, Chulalongkorn University, 254 Phayathai Road, Pathum Wan, Bangkok, 10500, Thailand, 66 8-860-318-88; 2 Department of Social and Administrative Pharmacy, Faculty of Pharmaceutical Sciences, Prince of Songkla University, Songkla, Thailand; 3 Department of Social and Administrative Pharmacy, Faculty of Pharmaceutical Sciences, Khon Kaen University, Khon Kaen, Thailand; 4 Food and Drug Administration Thailand, Nonthaburi, Thailand

**Keywords:** financial, economics, R&D, research and development, surveys, interviews, costs, revenue, policies, drugs, pharmaceuticals

*This is the authors’ response to peer-review reports for “Financial Feasibility of Developing Sustained-Release Incrementally Modified Drugs in Thailand’s Pharmaceutical Industry: Mixed Methods Feasibility Study.”*

## Round 1 Review

Thank you for your valuable comments and for recognizing the importance of conducting incrementally modified drug (IMD studies. We appreciate your feedback and have made the necessary revisions to improve the clarity, depth, and quality of the paper [1]. Below are our responses to each point.

### Reviewer H [2]

#### General Comments

*This paper provides valuable insights into how the Thai pharmaceutical industry should prepare for future developments. The results can be used as a reference to support decision-making and to guide the definition of regulations and processes in Thailand.*

#### Specific Comments

##### Major Comments

*1. Methods: Could you elaborate on how the 5 incrementally modified drug (IMD) experts were selected? Additionally, why was the number of experts limited to 5?*

**Response**: Thank you for your insightful question. We conducted in-depth interviews with 15 participants, ensuring data saturation in accordance with qualitative research methodology. Among them, 5 were local company owners specializing in IMD development, as they provided firsthand insights into industry challenges and opportunities. The remaining participants included experts from various sectors of IMD advancement, such as regulatory affairs, financial modeling, and clinical development, ensuring a comprehensive and diverse perspective. The selection criteria were designed to capture a balanced representation of stakeholders in the IMD landscape. Relevant details are provided in lines 101‐102.

*2. Tables 1 and 2: Please replace the term “Literature Review” with the specific author names and the corresponding year (Anno Domini).*

**Response**: Thank you for your suggestion. We have replaced the term “literature review” with the specific author names and corresponding year where applicable. However, for sources derived from government documents and institutional reports, we have used the official abbreviations of the respective organizations to maintain clarity and accuracy.

*3. Table 3: The values of US $1.46 million and US $18.6 million refer to the research and development costs only, correct? These values do not reflect the total cost of developing IMDs (refer to Table 2).*

**Response**: Thank you for your inquiry regarding the values listed in Table 3. To clarify, the figures of US $1.46 million and US $18.6 million indeed represent comprehensive cost assessments. These values encompass the entirety of the research and development expenditures, which includes formulation development, clinical trials, production batches necessary for registration, and the registration process itself. The provided values are intended to reflect the total cost incurred up until the point of market authorization. We have ensured that these costs cover most, if not all, expenses associated with the development of IMDs before reaching market readiness. This clarification has been detailed in Table 2.

*4. Since most of the numbers come from expert input, how do you ensure that these numbers are valid and accurately reflect real-world situations? It may be helpful to provide more information about the characteristics and qualifications of the key informants to support their credibility.*

**Response**: Thank you for your thoughtful comment. To ensure the validity and real-world accuracy of expert-provided data, we applied a triangulation approach, incorporating insights from multiple sources, including literature reviews, surveys, and interviews. This cross-verification process enhanced the consistency and reliability of the findings. Additionally, the experts were selected based on their extensive experience and qualifications in drug development. They include industry leaders, policy makers, and researchers with direct involvement in IMD development and financial modeling. The relevant details can be found in lines 80‐84 and 101‐102. Please let us know if further clarification is needed.

##### Minor Comments

*5. Please ensure that all abbreviations are defined the first time they appear in the document. For example, “IMD” should be written out as “Innovative Medical Devices (IMD)” when it is first mentioned, particularly in the introduction.*

**Response**: Thank you for your feedback. We have reviewed the document and ensured that all abbreviations are properly defined upon first mention.

### Reviewer BK [3]

#### General Comments

*This paper presents a thorough analysis of the financial feasibility of developing incrementally modified drugs (IMDs) within the Thai pharmaceutical industry. It aligns well with Thailand’s National Strategic Master Plan and provides valuable insights for stakeholders regarding investment decisions and policy development. The mixed- methods approach, including financial modeling, surveys, and interviews, lends credibility to the findings, while the focus on sustained-release dosage forms highlights a specific and practical application. The paper is well- structured and contributes meaningfully to the discussion on enhancing local pharmaceutical capabilities. However, there are areas where clarity, presentation, and depth can be improved to strengthen its impact.*

#### Specific Comments

##### Major Comments

*1. Clarity in objectives: While the paper provides an extensive background on Thailand’s pharmaceutical landscape, the research objectives could be more explicitly stated at the beginning of the introduction to guide the reader more effectively.*

**Response**: Thank you for your suggestion to enhance the clarity of the research objectives. We have revised the introduction to clearly and explicitly state the research objectives at the beginning, providing better guidance for the reader and improving the overall clarity of the study’s purpose.

*2. Discussion of results: The discussion section could delve deeper into comparing the financial feasibility of IMDs with other pharmaceutical products, especially generic drugs, to highlight the broader implications of the findings.*

**Response**: Thank you for your valuable suggestion on comparing IMDs with other pharmaceutical products. We have expanded the discussion section to provide a more in-depth comparison of the financial feasibility of IMDs with new drugs, new generic drugs, and the US Food and Drug Administration 505(b)(2) New Drug Application program, enhancing the applicability of the findings. The revisions can be found in lines 191‐199.

*3. Policy recommendations: Although the paper suggests policy recommendations, it would benefit from providing concrete examples of how these policies have been successfully implemented in other regions or industries. This would add depth and context to the recommendations.*

**Response**: Thank you for your valuable feedback on the policy recommendations section of our manuscript. We acknowledge your suggestion to enhance this section by providing concrete examples of successful policy implementations from other regions or industries. However, given the primary focus of our study on the financial aspects of developing IMDs within Thailand’s pharmaceutical industry, we have revised the manuscript to refine the scope of our conclusions. In this revision, we have removed detailed policy recommendations. Instead, we now suggest that the findings could be beneficial for planning strategic support within the industry. This adjustment helps to maintain the focus on the financial analysis and ensures that the recommendations are directly supported by our research findings without extending beyond the evidence provided. We believe this approach will keep the study concise and focused on its primary objectives.

*4. References and citation quality: The paper relies on only 15 references, which is insufficient for a study of this scope. Furthermore, only a few of these references are from peer-reviewed scientific journals, while the rest are reports and secondary sources. This significantly weakens the academic foundation of the study. It is strongly recommended to update the references section by incorporating recent, high-quality, and peer-reviewed articles.*

**Response**: Thank you for highlighting this weakness in our study. We have strengthened its academic foundation by incorporating additional high-quality, peer-reviewed articles. However, as IMD remain a relatively new topic with limited peer-reviewed literature available, we primarily relied on in-depth interviews as the main methodology for estimating costs and key parameters.

##### Minor Comments

*5. Terminology consistency: Terms like “incrementally modified drugs” and “IMDs” should be consistently used throughout the text to avoid confusion.*

**Response**: Thank you for your feedback. We have reviewed the document and ensured that all abbreviations are properly defined upon first mention.

*6. Figures and tables: Ensure all figures and tables are adequately labeled and referenced in the text. For instance, the presentation of financial data could be enhanced with clearer visualizations.*

**Response**: Thank you for your valuable suggestion. We have revised all three tables for improved clarity and ensured that they are properly referenced throughout the text.

*7. Formatting and grammar: Minor grammatical errors and formatting inconsistencies (eg, use of citations and spacing) should be addressed for a polished presentation.*

**Response**: Thank you for highlighting this point. We have carefully reviewed the document to correct formatting inconsistencies, improve citation accuracy, and ensure grammatical correctness.

*8. Abstract refinement: The abstract could be more concise, emphasizing key findings and policy implications without overly detailed descriptions of methods.*

**Response**: Thank you for your feedback. We have revised the abstract into a structured format, making it more concise while emphasizing key findings.

*9. Future research directions: Including a section on future research directions would enhance the paper’s utility for academics and policy makers.*

**Response**: Thank you for your valuable feedback on future research directions. As we mentioned earlier, IMDs are relatively new, presenting numerous research opportunities. In response, we have added a future research directions section, offering insights into the development of IMDs from patient, regulatory, and market-access perspectives. This addition provides valuable data for policy makers and the industry. The revisions are reflected in lines 216‐223.

We appreciate the detailed feedback, which has significantly improved the clarity, structure, and academic rigor of our study. Please let us know if further refinements are needed.

## Abbreviations

IMD: incrementally modified drug
